# History of Parkinson’s Disease-Associated Gene, *Parkin*: Research over a Quarter Century in Quest of Finding the Physiological Substrate

**DOI:** 10.3390/ijms242316734

**Published:** 2023-11-24

**Authors:** Tohru Kitada, Mustafa T. Ardah, M. Emdadul Haque

**Affiliations:** 1 Otawa-Kagaku, Parkinson Clinic and Research, Kamakura 247-0061, Japan; tohrukitada@gmail.com; 2 Department of Biochemistry and Molecular Biology, College of Medicine and Health Sciences, United Arab Emirates University, Al Ain P.O. Box 15551, United Arab Emirates; mustafa_ardah@uaeu.ac.ae; 3 Zayed Center for Health Sciences, United Arab Emirates University, Al Ain P.O. Box 15551, United Arab Emirates

**Keywords:** Lewy bodies, Parkinson’s disease, Parkin, PINK1

## Abstract

*Parkin*, the gene responsible for hereditary Parkinson’s disease (PD) called “Autosomal Recessive Juvenile Parkinsonism (AR-JP)” was discovered a quarter of a century ago. Owing to its huge gene structure and unique protein functions, *parkin* has become a subject of interest to those involved in PD research and researchers and clinicians in various fields and is being vigorously studied worldwide in relation to its nature and disease. The gene structure was registered under the gene name “*parkin*” in the GenBank in 1997. In 1998, deletion and point mutations in the *parkin* gene were reported, thereby demonstrating *parkin* is the causative gene for hereditary PD. Although 25 years have passed since the gene’s discovery and many researchers have worked tirelessly to elucidate the function of the Parkin protein and the mechanism of its role against neuronal cell death and pathogenesis remain unknown, which raises a major question concerning the current leading hypothesis. In this review, we present the results of related research on the *parkin* gene in chronological order and discuss unresolved problems concerning its function and pathology as well as new trends in the research conducted to solve them. The relationship between *parkin* and tumorigenesis has also been addressed from the perspective of Parkin’s redox molecule.

## 1. Cloning of *Parkin* Gene in a Short Period: Three Lucky Breaks

In the late 1990s, the global sequencing teams in the Human Genome Project were still in the process of deciphering the entire genome sequence, while the genomic information itself remained unclear.

First, a polymorphism analysis was performed for the manganese superoxide dismutase (Mn-SOD), a mitochondrial enzyme that has an antioxidant effect, as a candidate gene for hereditary Parkinson’s disease (PD). In one patient’s family, all patient-specific genetic polymorphisms were observed [1], and, ultimately, it became clear that the *Mn-SOD* was not the causative gene. Nevertheless, this linkage analysis result convinced that the causative gene was located near the *Mn-SOD* gene, which was the first lucky break. Among the 46 chromosomes, it was possible to concentrate on linkage analysis of the long arm of chromosome 6, where the *Mn-SOD* gene resides.

In the second lucky break, following a series of linkage analyses, the microsatellite marker D6S305, located on the long arm of chromosome 6, was linked to the patient’s pedigree, and the patient was found to lack this marker. Thus, it was found that the causative gene exists in the gene region in chromosome 6 containing D6S305. Nevertheless, identifying the causative genes from hundreds of genes ranging from the 3 Mb is important. Exons were searched from the BAC DNA library using the exon trap method with the D6S305 as a probe; however, only a few exons were identified.

Ultimately, *parkin* was the only gene in the vicinity and was found to be an unknown jumbo gene of >1 Mb, consisting of only 12 exons. Due to this, the reason why exon trapping can identify only a few exons became clear. This was the third time lucky, as it was enough to end up with only one genetic analysis.

Thus, a gene that is second in size to the muscle disease gene *dystrophin* (approximately 2.5 Mb), which was previously known as a giant gene, has been discovered. Kitada proposed the name “*parkin*” to represent the primary disease, and, above all, he hoped that the discovery of this gene could promote pioneering research on the etiology of PD [2]. Kitada et al. (1998) also reported that mutations of the *parkin* gene are associated with autosomal recessive juvenile parkinsonism (AR-JP). Important findings related to the *parkin* gene are summarized in Table 1.

## 2. Why Abnormal Mitochondrial Accumulation Theory Has Been Supported for So Long: Three Turning Points

### 2.1. The First Turning Point—Year 2000

The first turning point was the proposal of the assumption that the etiology of the AR-JP is a high intracellular accumulation of substrate proteins due to *parkin* deficiency. This assumption is in direct conflict with the pathological reports of pathologists and neurologists [16,17,18].

**Figure 1:**
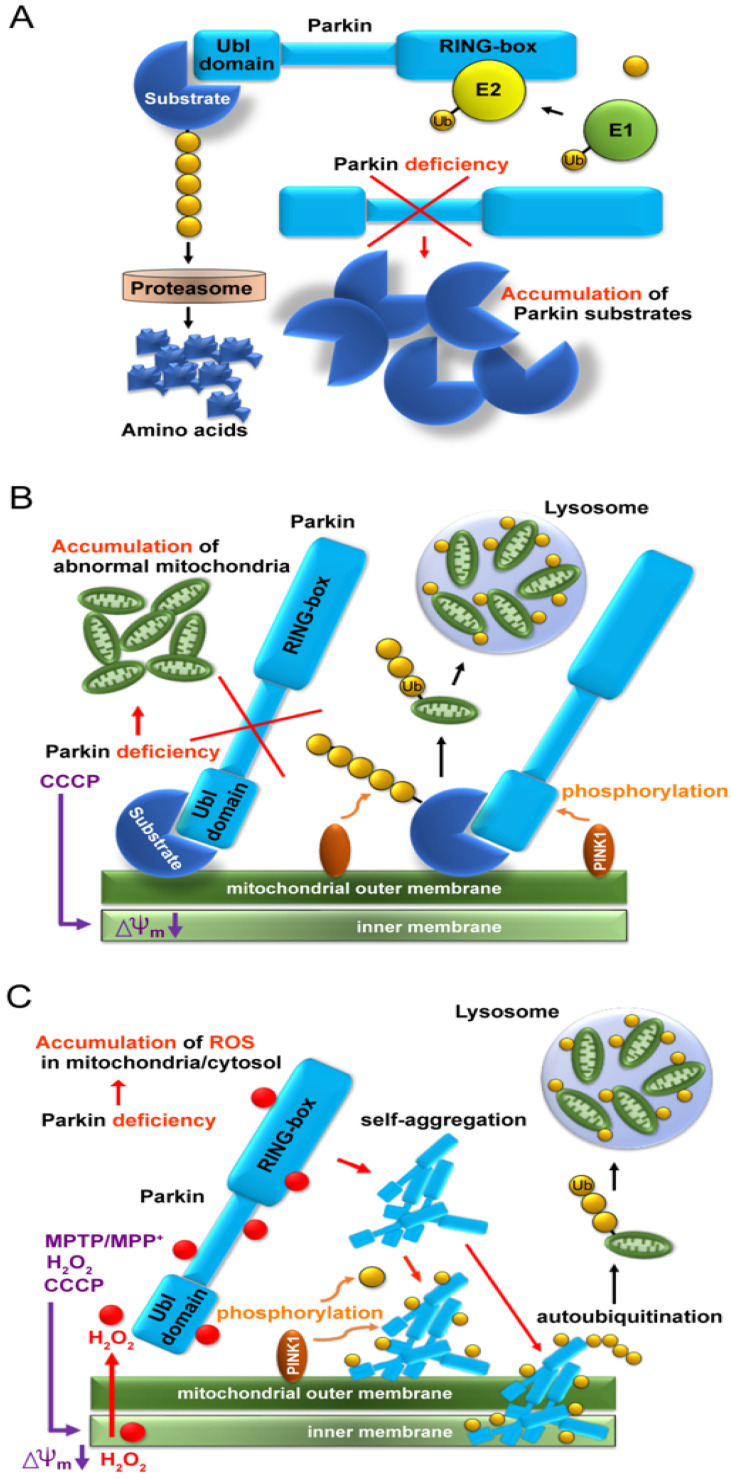
Schema of three leading hypotheses. (**A**) Accumulation of substrate proteins due to lack of proteasomal degradation caused by *parkin* deficiency. (**B**) Accumulation of aberrant mitochondria due to failure of mitochondrial ubiquitination caused by *parkin* deficiency. (**C**) Accumulation of aggregated and insoluble Parkin proteins in mitochondria due to reaction with hydrogen peroxide leaked from aberrant mitochondria.

Since the Parkin protein was recognized as an E3 ligase in 2000 [5], a reasonable but simple idea was raised that Parkin must have substrates and its deficiency would cause their accumulation due to proteasomal failure (Figure 1A). Subsequently, similar to bamboo shoots in rain, countless candidates for Parkin’s substrates have been reported over approximately 10 years. However, neither the characteristic structure nor function common to these candidates has been reported, nor has the high accumulation of specific proteins been pathologically explained or identified.

### 2.2. Genetic Interaction between Parkin and PINK1 Genes: Knowledge Gathered from Fruit Fly Models

In 2004, the *PTEN-induced kinase 1* (*PINK1*) gene was identified as the third causative gene of autosomal recessive Parkinson’s disease (ARPD) [6]. The PINK1 protein is thought to be a serine/threonine protein kinase localized in the mitochondrial inner membrane. Recent in vitro and cultured cell studies have reported that PINK1 phosphorylates and activates Parkin and ubiquitin [19].

In 2006, genetic studies on the Drosophila melanogaster reported that the PINK1 functions upstream of Parkin. The *parkin*- and *PINK1*-deficient Drosophila melanogaster has been observed to exhibit flight muscle degeneration, short lifespan, male sterility, and decreased energy [8,9]. Overexpression of the *PINK1* in *parkin* knockout flies did not improve the above phenotype; however, the *parkin* overexpression did improve the phenotype in the *PINK1* knockout flies.

### 2.3. The Second Turning Point—Year 2008

Biological cell research on the Parkin flourished in the years leading up to 2008, when a surprising phenomenon was reported in which the Parkin localized defective mitochondria with reduced membrane potential, leading to mitochondrial degradation via autophagy called “mitophagy” [10].

In 2010, two groups demonstrated a cooperative relationship between Parkin and PINK1 in abnormal mitochondrial processing (Figure 1B) [11,12]. When the mitochondria are artificially depolarized using a mitochondrial uncoupler, such as carbonyl cyanide m-chlorophenyl hydrazone (CCCP), after the overexpression of the tagged Parkin in cultured cells, PINK1 recognizes depolarization and moves from the inner membrane to the outer membrane, where it recruits Parkin, which polyubiquitinates various mitochondrial substrates and induces mitophagy. Thus, defects in Parkin or PINK1 are thought to prevent the degradation of these abnormal mitochondria, which accumulate intracellularly and cause neuronal cell death. The substrates on the mitochondria themselves do not appear to be defective; however, Parkin ubiquitinates them non-specifically in depolarized mitochondria.

One of the two groups that presented the results argued for approximately 10 years that Parkin deficiency failed to degrade the Parkin substrates and that the accumulation of those substrates caused neuronal cell death [5]. In this case, specimens from patients with AR-JP should have been thoroughly examined for abnormal mitochondrial deposition using a variety of orthodox staining methods, such as hematoxylin-eosin (HE) staining, protein staining, or electron microscopy.

More than a decade later, the intracellular number of abnormal mitochondria, their morphology, and tissue distribution remained unknown. In contrast to the beta-amyloid deposits found in Alzheimer’s disease and alpha-synuclein deposits seen in sporadic PD, the inability to pathologically diagnose accumulated abnormal mitochondria is paradoxical.

In conclusion, the primary problem with this hypothesis is that it cannot be confirmed pathologically or histologically. Unfortunately, we used the same route as before. No abnormal mitochondrial accumulation has been reported in autopsy or biopsy cases of the *parkin* or *PINK1* mutant patients. Details are described in the clinical and pathological aspects of AR-JP.

### 2.4. The Third Turning Point: “Parkin Dilemma”

An important functional and structural feature of the Parkin protein has been neglected, which is the third turning point (unfortunate) of Parkin research. The Parkin protein contains 35 cysteine residues (7.5%) out of 465 amino acids. Such cysteine-rich proteins are rare among the E3 ligases. The cysteine residue contains a thiol group (-SH) that scavenges superoxide (O_2_^−^) and hydrogen peroxide (H_2_O_2_) and forms a disulfide bond (S-S). It was observed that Parkin reacts directly with and reduces H_2_O_2_ and aggregates into dimers and multimers, resulting in poor solubility [14,20,21].

When CCCP is used to collapse the inner membrane potential of mitochondria, the membrane potential of all mitochondria decreases, and ions, proteins, and reactive oxygen species (ROS) leak into the cytoplasm through the collapse. It is extremely disappointing that many researchers claiming mitochondrial ubiquitination and mitophagy deficiency due to the lack of Parkin or PINK1 have not taken into account Parkin’s function as a redox molecule and its secondary consequences. The E3 ligase function of Parkin and its function as a redox molecule are essential, but not simultaneous. Thus, when Parkin scavenges H_2_O_2_ as a redox molecule, it aggregates and loses E3 activity, which is called the “Parkin dilemma”.

## 3. Clinical and Pathological Aspects of *Parkin* (AR-JP, PARK2) and *PINK1* (PARK6) Mutations

The focus here was on studying the PINK1/Parkin pathway using molecular and cell biological techniques, and the clinical and pathological aspects of the two autosomal recessive Parkinson’s Disease (ARPDs) were referred to. First, the two inherited diseases are genetically independent. 

Compared to the sporadic form, its prominent clinical feature is Parkinsonism with marked diurnal fluctuations. In addition, AR-JP has a generally benign clinical course, with marked effects of levodopa, high frequency of dyskinesia and dystonia, and hyperreflexia. If present, autonomic symptoms are mild [3,4,16,17,18]. AR-JP autopsy studies typically show the loss of neurons without Lewy bodies, gliosis, relatively localized lesions in the substantia nigra and locus coeruleus, and the presence of low or immature melanin in residual neurons. However, several cases of Lewy bodies have also been reported [22,23].

In contrast to the AR-JP phenotype, patients with *PINK1* mutations rarely present with symptoms specific to patients with AR-JP described above. Families with *PINK1* (PARK-6) mutations have a wide age range of onset (up to 68 years) and slow progression [24]. Features typical of AR-JP, such as dystonia at onset and sleep effects, are not observed in PARK6-related families; thus, the clinical manifestations of late-onset cases are indistinguishable from those of sporadic PD [24]. However, sporadic PD with heterozygous variants of *PINK1* has been reported to overlap with some of the characteristic clinical features of AR-JP and should be noted [25].

Pathologically, as of 2020, three autopsy brain reports of *PINK1* gene mutations have been reported, including three patients with Lewy-related pathology and one patient without Lewy-related pathology [26]. Thus, it cannot be concluded that Parkin and PINK1 are involved in the same pathway in both clinical and pathological aspects.

To date, there have been many reports of brain autopsies or biopsies of tissues of *parkin* mutants and several reports of brain autopsies of the *PINK1* mutants. However, none of them exhibited abnormal mitochondrial accumulation. There are hundreds to thousands of mitochondria in a single cell, and they have a very short lifespan of seven to 10 days. If these mitochondria are not processed, thousands of unnecessary mitochondria can accumulate in just one week, and many abnormal mitochondria can be expected to accumulate in the cell every day.

In the Parkin/PINK1 pathway, PINK1 is upstream of Parkin, and the current theory is that the phosphorylation of Parkin by PINK1 is essential for Parkin recruitment. However, this contradicts the results of the two fruit fly studies. The phenotype of *PINK1*-deficient fruit flies should be recovered by overexpression of *parkin* [8,9].

However, the two studies described below have reported muscle biopsies of patients with AR-JP [27,28,29]. Although two studies on fruit flies, the starting point of the Parkin/PINK1 pathway theory, acknowledged significant findings, only minor changes in muscle fibers were observed histologically. These reports are valuable because mitochondria undergo progressive degeneration in post-mortem specimens. Tissue biopsies can provide a tissue environment similar to that of the living environment.

In an electron microscopy study by van der Merwe et al., muscle biopsies were obtained from two patients [27]. Muscle fibers showed subtle abnormalities such as slightly swollen mitochondria in the focal areas of the fibers and some folding of the sarcolemma.

The laboratory of Murat Emre at Istanbul University performed immunohistochemistry on biceps brachii muscles from three patients with exon 9/intron 9 junction “G” deletion or exon 3 deletion of the *parkin* gene and observed only mild histopathological changes in the muscles [28]. Researchers have also reported a patient with a homozygous IVS-9-1 deletion in the *parkin* gene [29]. The patient had bilateral thigh muscle hypertrophy and a muscle biopsy of the biceps revealed abundant cytochrome oxidase (COX) (-) fibers.

Nevertheless, only minor pathological changes have been reported in muscle biopsies of *parkin* mutants at both electron and light microscopic levels, and abnormal mitochondrial accumulation has not been reported.

## 4. Studies Focusing on Parkin as a Redox Molecule Alongside E3 Ligases

In the early 2000s, Parkin was found to be oxidized to form aggregates [7]. Meng et al. showed that oxidative stress causes sulfation/sulfonation of cysteine-rich regions of Parkin, resulting in decreased E3 activity [13]. In addition, since the discovery of the *parkin* gene, numerous chemical and histological reports of increased oxidative stress in the *parkin*-knockout cultured cells and animal models have been reported [30,31]. Furthermore, it has been reported that iron staining in the substantia nigra of AR-JP is more intense than in controls or sporadic PD, suggesting that oxidative stress may play an important role in the neurodegeneration that occurs in AR-JP [32]. Recently, Tokarew et al. have reported a series of Parkin protein analyses using the following human autopsy brains [21]. First, Parkin solubility is decreased in aged human brains, including the substantia nigra. Decreased Parkin solubility is correlated with increased levels of hydrogen peroxide in human brains. They also demonstrated elevated hydrogen peroxide in the AR-JP brain. While it has been reported that Parkin reacts with dopamine and its metabolites and reduces their toxicity [7]. Tokarew et al. have also experimentally shown a protective role for Parkin as a redox molecule, including conjugation of reactive dopamine metabolites, containment of radicals within insoluble aggregates, and increased melanin formation [21]. At the beginning of this review, hypoplasia of neuromelanin in the substantia nigra was mentioned as a characteristic pathological finding of AR-JP. From these findings, it is understood that neuromelanin formation is not successful without Parkin. The biochemistry and pathology of AR-JP thus accumulated are proving the importance of Parkin’s function as a redox molecule, and the contradictory pathological findings are not within our knowledge.

The precise mechanism by which the Parkin protects itself from oxidative stress has long been unclear. Kitada et al. proved that Parkin reacts directly with hydrogen peroxide and reduces it significantly in a very simple experiment in which Parkin protein and hydrogen peroxide solution were mixed in a test tube [14]. Until then, Parkin was perceived negatively because its E3 function was impaired by oxidative stress, but another important function, the antioxidant effect, of Parkin is now being recognized. However, these two aspects cannot exist simultaneously, and this fact should be called the “Parkin dilemma”, as mentioned above.

After overexpression of various combinations of Parkin, PINK1, and Ubiquitin in HEK293 cultured cells for 24 h and CCCP treatment, mitochondrial fractions were extracted and Western blot (WB) analysis was performed using a reducing agent-free loading dye [15]. Surprisingly, the E3 Parkin monomer was not recruited into the mitochondria by PINK1 but was deposited as aggregates on the outer and inner mitochondrial membranes and was self-ubiquitinated (auto-ubiquitination). Rather than phosphorylating Parkin monomers to activate them, PINK1 seemed to phosphorylate Parkin aggregates of various sizes and promote Parkin aggregation, making them insoluble (Figure 1C). Interestingly, when WB was performed using a loading dye containing the reducing agent (dithiothreitol [DTT]), the poorly soluble Parkin aggregates were broken down to the monomer level. When cultured cells were treated with glutathione before adding CCCP, some bands at the monomer level were observed even when WB was performed using DTT-free loading dye. These results suggest that at the cellular level, glutathione, which is the most abundant reducing substance in the body, partially converts insoluble Parkin into an E3 monomer. Intracellular regulation between Parkin aggregates and Parkin monomers needs to be investigated in the future.

At the beginning of this section, we introduced the concept that Parkin, as a redox molecule, can effectively explain the pathology of Autosomal Recessive Juvenile Parkinsonism (AR-JP). However, research into Parkin’s role as a redox molecule is still in its early stages, and much remains to be discovered about its key functions and the impacts of its deficiency. The Parkin protein contains 35 cysteine residues, but which of these are actively involved in redox reactions? Can we identify Parkin point mutations that impair its ability to bind to H_2_O_2_? Therefore, a detailed investigation from multiple angles is required. This includes determining if these point mutations align with those found in AR-JP patients.

## 5. Important and Noteworthy Key Points Derived from the Experiments

Herein, the learnings and experience gained from experiments conducted by Ardah MT and colleagues using Parkin, which has two roles: an E3 ligase and a redox molecule, are listed. This study is expected to contribute to the overall understanding of Parkin function, the pathogenesis caused by *parkin* deficiency, and the mechanism of neuronal death (and tumorigenesis).

### 5.1. Understanding That Different Cell Lines Were Used to Produce Different Processes and Results

Most cell biological studies on the Parkin/PINK1 pathway have used HeLa cells, a cell line established from cervical cancer cells, which are easy to culture and introduce genes into and have been widely used in cell biology research. However, it is questionable whether this method is suitable for studying normal mitochondria. Various cancer cells have reduced mitochondrial function and produce energy mainly through the glycolytic system, despite the presence of sufficient oxygen. This is known as the “Warburg effect” and is a characteristic of cancer. Ardah et al. used HEK293 cells, a cell line derived from the human fetal kidney, which is also tissue culture-expandable and easy to transfect [15]. In both cases, the amount of endogenous Parkin and PINK1 was very small, and it was difficult to study their dynamics and morphological changes without overexpressing the tagged *parkin* and *PINK1* in cultured cells.

SH-SY5Y cells are derived from neuroblastoma cell lines. The SH-SY5Y cells are often used as a model for in vitro studies of neuronal function and differentiation and have also been used to study PD because they produce dopaminergic markers. To avoid artifacts caused by artificially expressed genes in cultured cells, Ardah et al. screened several representative cell lines to obtain those that could produce an appropriate amount of endogenous Parkin [15]. The SH-SY5Y cell line was found to express both Parkin and PINK1 more abundantly than other cell lines.

Thus, they reported a new finding using the SH-SY5Y cell lines: PINK1 is stably expressed in the mitochondria without CCCP administration [15]. The prevailing hypothesis is that after the translocation of PINK1 to the mitochondrial inner membrane, the N-terminus of PINK1 is cleaved by presenilin-associated rhomboid-like protein and mitochondrial processing peptidase [33,34], and PINK1 is transported back to the cytoplasm and degraded. When the membrane potential decreases, the N-terminus of PINK1 is not cleaved, and it accumulates in the mitochondrial outer membrane. However, in the study using the SH-SY5Y cells, almost equal amounts of PINK1 were observed in a WB of mitochondrial fractions with and without CCCP [15]. It should be noted that the different cell lines yielded different results. In cells such as the SH-SY5Y, where the ROS tends to accumulate in the mitochondria and cytoplasm, PINK1 seems to constantly perform some functions in the mitochondria. Previously, reduced mitochondrial respiratory capacity, increased mitochondrial calcium ions, and oxidative stress in the *PINK1*-deficient mouse brains, as well as increased oxidative stress in the cytoplasm, have been observed [35]. In contrast, the assessment of mitochondria using electron microscopy in *PINK1*-deficient mouse brains showed no significant changes in mitochondrial structure or number, although the mitochondria in the *PINK*1-deficient mice were slightly larger in size.

Finally, in 2006, Yang et al. showed that the exposure of the SH-SY5Y cell line to the oxidant dopamine upregulated *parkin* expression. They performed a luciferase assay and found that a specific *parkin* gene promoter construct promoted transcriptional activation in response to dopamine, and attributed *parkin* upregulation to transcriptional activation [36]. This effect was confirmed when the SH-SY5Y cells were exposed to another oxidative stress, MPP+ (a metabolite of MPTP).

### 5.2. The Uncoupler CCCP Can Cause Unexpected Artifacts

The use of mitochondrial inner membrane uncouplers, including CCCP, is an experimental manipulation that is a major pillar of the Parkin/PINK1 pathway hypothesis. Using this process as a membrane potential lowering model, they explained the operation with a simple phrase: “When the membrane potential is lowered by CCCP”. However, this chemical is a powerful drug, and its effect should not be described as a reduction in membrane potential. It causes all the internal ions, proteins, and ROS from every mitochondrion in the cell to leak into the cytoplasm in one fell swoop. It is not difficult to predict that countless molecules, not just Parkin and PINK1, can exhibit unpredictable behaviors. They can also produce various artificial artifacts. A rapid rise in calcium ions and changes in cellular pH can interfere with the series of E1, E2, and E3 reactions (Parkin) in the ubiquitin-proteasome system, and a rapid increase in cytoplasmic H_2_O_2_ can consume Parkin proteins and form insoluble Parkin aggregates.

### 5.3. Reducing Agents in the Loading Dye for WB Degrade Parkin Aggregates

Among the numerous experiments testing the conventional theory, the most overlooked issue regarding experimental manipulation is whether the loading dye contains a reducing agent when performing a WB.

The Parkin was overexpressed in cultured cell lines, CCCP was administered 24 h later, mitochondrial fractions were extracted, and WB was performed under reducing conditions using a loading dye containing the DTT and under non-reducing conditions without it. The results showed that the DTT instantly cleaved the S-S bonds and degraded high-molecular-weight (HMW) Parkin aggregates to monomeric levels. At first, the WB under reducing conditions was observed to have a Parkin monomer with E3 activity; however, WB under non-reducing conditions showed that actual Parkin exists as ubiquitinated HMW aggregates. Thus, rather than being recruited by PINK1, the Parkin monomers reacting with H_2_O_2_ were deposited on the mitochondria. They appeared to be aggregated and ubiquitinated.

### 5.4. Pitfalls Associated with Tagged Parkin Overexpression

The observation of intracellular behavior by tagging a gene product of interest with a fluorescent protein has been established as a modern cell-biological technique and is frequently utilized. This technique allows us to observe the behavior of specific proteins in living cells. However, it should be noted that this can also alter the original nature of these proteins and introduce artifacts into the experiments.

The laboratory of Helen Walden at The London Research Institute of Cancer Research has characterized the Parkin proteins with various tags in detail [37,38] through cell culture and in vitro studies. They showed that the ubiquitin-like domain of the Parkin functions to inhibit its self-ubiquitination, and that pathogenic *parkin* gene mutations in this domain disrupt this autoinhibition, resulting in a constitutively active molecule.

They observed that the large MBP-, GST-, or SUMO-tagged Parkin was constitutively active, suggesting a disruption of the autoinhibitory state. They also reported that small epitope tags, such as the Myc, FLAG, and HA peptide tags fused to the N-terminus of Parkin, also disrupted the Parkin autoinhibition and stability. Furthermore, the mechanism of this autoregulation involves ubiquitin binding via the C-terminal domain of Parkin.

In fact, when the WB was performed on the cultured cells overexpressing GFP- and HA-*parkin*, it was observed that the GFP-Parkin more strongly appeared as ubiquitinated HMW aggregates [15].

## 6. Role of Parkin―Mitochondrial Oxidative Stress―Mitophagy―Cancer

A link between Parkin and tumorigenesis has been previously noted. At least two studies on the relationship between these two were published in 2003.

Denison et al. described the FRA6E (6q26) as the third most frequently observed common fragile site (CFS) in human chromosomes, and the *parkin* gene (PARK2) accounts for more than half of this CFS [39]. The PARK2 expression was downregulated in 60.0% of primary ovarian tumors analyzed using the RT-PCR. Loss of heterozygosity (LOH) analysis of primary ovarian tumors using polymorphic markers in the 6q26 region showed 72% LOH in the center of PARK2, which was the highest among the markers tested. The authors concluded that the FRA6E represents a large region of genomic instability and contains very large genes that may play a role in the development of ovarian and other cancers.

Cesari et al. also reported that the LOH analysis of 40 breast and ovarian cancers identified a common minimally defective region containing the markers D6S305 (50%) and D6S1599 (32%), both within the PARK2 [40]. *Parkin* gene expression appeared to be reduced or absent in tumor biopsies and tumor cell lines. These data also suggest that the LOH observed on chromosome 6q25-q26 may contribute to cancer initiation or progression by inactivating or reducing *parkin* gene expression. Since then, there have been a series of reports on Parkin’s tumor-suppressive function in various carcinomas and as a factor in cancer aggravation due to its deletion; however, details regarding its molecular mechanism are still unknown. Recently, however, several review articles have been published describing considerations regarding this mechanism [41,42,43], A summary is presented below. In each case, the proposed mechanism is based on results obtained in terms of E3 Parkin function, and the substrates or *parkin* regulators that may be involved in the mechanism are noted in parentheses [41]: cell cycle (Cyclin E), proliferation (Cyclin B1 and Aurora A/B), apoptosis (Mcl-1, a Bcl-2 family member), migration/invasion and metastasis (HIF-1 alpha), metabolic reprogramming (p53 and PKM 2), mitophagy (PINK1).

According to Denisenko et al., similar to autophagy, mitophagy plays a dual role in cancer and may promote or suppress tumorigenesis, depending on the tumor type and molecular context. The loss of function of several mitophagy-related genes inhibits mitophagy and causes further accumulation, which causes mitochondrial dysfunction and contributes to tumorigenesis. In contrast, mitophagy may function as a tumor-promoting mechanism that contributes to cancer cell survival under stressful conditions [43].

However, the following two points should be emphasized in this section: First, there are no statistical reports or evidence that patients with AR-JP have a higher incidence of any cancer. Although the number of patients with AR-JP is high among familial PD, the absolute number is very small. Nevertheless, mutations in the *parkin* gene are found at high frequencies in various cancer types, and *parkin* is present in three major CFSs, making it genetically vulnerable. Given this, if cancer cell escapes the immune system, they begin to proliferate indefinitely after developing in the human body. Thus, it is not difficult to imagine that, after the cancer cell starts proliferating, a mutation can occur in the mega-sized *parkin* gene on the long arm of chromosome 6, which is a genetically vulnerable site, and cancer cells with this genetic mutation can occupy a predominant position. Alternatively, it is possible that *parkin* gene mutations can occur first, and the subsequent increase in intracellular ROS can lead to cytotoxicity and DNA damage, resulting in oncogenesis.

Second, research reports and review articles have largely ignored the redox molecular aspect of Parkin. It was shown that Parkin, an excellent redox molecule, directly reacts with hydrogen peroxide, which constantly leaks from the mitochondria. At the same time, Parkin accumulates in the mitochondria and becomes insoluble. Haploinsufficiency of the *parkin* gene leads to an increase in mitochondria-derived ROS in cells and a decrease in mitochondrial function. Is this consequence beneficial to cancer cells?

Cancer cells establish their own cancer metabolic networks by rearranging their metabolic pathways to aid in cellular carcinogenesis and malignant transformation. For example, cancer cells are known to produce ATP through glycolysis rather than mitochondrial oxidative phosphorylation, even under aerobic conditions, which is called the Warburg effect and is thought to be an adaptation to a hypoxic environment. However, under hypoxic conditions, the lack of oxygen for accepting electrons causes inefficient electron transfer in the mitochondrial electron transport system, resulting in ROS accumulation. These cells adapt to hypoxia via hypoxia-inducible factor 1 (HIF-1), which triggers various molecular events [44]. It is speculated that intracellular ROS levels are further elevated in an environment lacking the redox molecule Parkin. Therefore, it is natural for cancer cells with mutations in the *parkin* gene to adapt to a hypoxic/high ROS environment and become dominant among cancer cells.

## 7. Conclusions

Although the above discussion is only an expectation, the purpose of this review is to emphasize that Parkin plays an extremely important role as an E3 ligase and a redox molecule. The structural biology of the E3 Parkin and its functional analyses have made remarkable progress and should be further explored in the future. However, we believe that the relationship between Parkin and carcinogenicity, as well as PD, should be reviewed from these two perspectives to elucidate the true pathophysiology and develop therapeutic methods. In addition, we should learn from Parkin’s work that in a medical hypothesis, it is essential to have parallel pathological and histological confirmation, rather than making a conclusion based only on molecular and cellular biological methods. Otherwise, the medical hypothesis can remain the only hypothesis forever.

## Figures and Tables

**Table 1:** Related clinical, pathological, and molecular findings on the Parkin are presented in chronological order.

Year	Summary of Research Findings	Source	References
1973	The first AR-JP family with a characteristic clinical picture was reported.	Yamamura Y et al., 1973	[3]
1993	The first brain pathology of AR-JP was reported, and nigral neuronal loss without Lewy bodies was observed.	Yamamura Y et al., 1993	[4]
1997	The AR-JP gene *parkin* was cloned, and the genetic information was formally registered in the GenBank.	GenBank accession #AB009973	[2]
1998	The discovery of the *parkin* gene and its various mutations were published.	Kitada T et al., 1998	[2]
2000	Parkin protein is recognized as an E3 ubiquitin ligase in the ubiquitin-proteasome system.	Shimura H et al., 2000	[5]
2004	Another autosomal recessive PD gene, *PINK1*, was identified.	Valente ME et al., 2004	[6]
2005	Dopamine covalently modifies Parkin in living dopaminergic cells, a process that increases Parkin insolubility and inactivates its E3 function.	LaVoie MJ et al., 2005	[7]
2006	Drosophila lacking *PINK1* gene showed similar phenotypes, but the overexpression of Parkin restored the defective symptoms caused by the loss of *PINK1*.	Clark IE et al., 2006	[8]
		Park J et al., 2006	[9]
2008	Parkin localizes to defective mitochondria with lowered membrane potential, leading to autophagy called mitophagy.	Narendra D et al., 2008	[10]
2010	Parkin is recruited by PINK1 on defective mitochondria, ubiquitinating outer membrane proteins and inducing mitophagy.	Narendra D et al., 2010	[11]
		Matsuda N et al., 2010	[12]
2011	Oxidation of the cysteine-rich region of Parkin reduces its E3 ligase activity and contributes to protein aggregation.	Meng F et al., 2011	[13]
2016	The Parkin protein reacts directly with and eliminates hydrogen peroxide.	Kitada T et al., 2016a	[14]
2023	Parkin reacts with hydrogen peroxide that leaks from mitochondria, causing self-aggregation and auto-ubiquitination on mitochondria.	Ardah MT et al., 2023	[15]

## Data Availability

Not applicable.
